# Intraoperative Peritoneal Lavage for Detection of Malignant Cells: Technique, Evidence, Clinical Relevance and Future Perspectives

**DOI:** 10.3390/cancers18101604

**Published:** 2026-05-14

**Authors:** Resa Puffert, Anna Quarder, Fabian Kockelmann, Thomas Wirth, Tanja Reineke-Plaaß, Mieke Raap, Moritz Schmelzle, Linda Feldbrügge, Beate Rau, Franziska Köhler

**Affiliations:** 1 Department of General, Visceral and Transplant Surgery, Hannover Medical School, Carl-Neuberg-Straße 1, 30625 Hannover, Germany; 2 Department of Gastroenterology, Hepatology and Endocrinology, Hannover Medical School, Carl-Neuberg-Straße 1, 30625 Hannover, Germany; 3 Department of Pathology, Hannover Medical School, Carl-Neuberg-Straße 1, 30625 Hannover, Germany; 4 Surgical Department, Charité-Universitätsmedizin Berlin, Corporate Member of Freie Universität Berlin and Humboldt Universität zu Berlin, 10117 Berlin, Germany

**Keywords:** peritoneal metastases, peritoneal lavage, intraoperative cancer staging, peritoneal cancer, peritoneal wash fluid

## Abstract

Background/Objectives: Peritoneal metastases represent a common manifestation of advanced gastrointestinal malignancies and are associated with poor survival. Their early detection is essential for adequate tumor staging, prognosis, and treatment selection, especially to avoid unnecessary surgery. Intraoperative peritoneal lavage has been established as a diagnostic tool to detect occult peritoneal disease. However, reported techniques, analytical methods, and detection rates vary considerably. The objective of this review was to summarize current approaches to intraoperative peritoneal lavage, evaluate different detection methods, and assess their clinical relevance. Methods: A literature search was performed using the PubMed database for studies published between 2015 and 2025. The search terms “intraoperative peritoneal lavage” or “peritoneal fluid cytology” were used. Studies were included if they evaluated peritoneal lavage as a diagnostic method for detecting malignant cells, including all primary tumors and disease stages. Articles focusing on lavage as a therapeutic intervention or lacking methodological details were excluded. Results: Physiological saline solution was used for lavage in all included studies, with volumes ranging from 10 to 1000 mL. Sampling was predominantly performed immediately after abdominal access in various abdominal sites. Detection methods varied widely, with conventional cytology being most frequently used, while molecular techniques were used in a smaller number of studies. Positive detection rates showed broad variations and were higher in advanced tumor stages. Conventional cytology showed limited detection rates compared to molecular approaches. Conclusions: Intraoperative peritoneal lavage remains a valuable but methodologically heterogeneous diagnostic tool with limited detection rates when relying on conventional cytology alone. Molecular techniques seem to improve the detection rate of occult peritoneal disease but require further standardization and validation before routine clinical implementation. The technique of peritoneal lavage should be standardized by implementing an international consensus including lavage sites, volume of applied fluid, and detection method.

## Simple Summary

Peritoneal metastases indicate advanced gastrointestinal cancer and are associated with poor outcomes. The early detection of cancer cells in the abdominal cavity is important for accurate staging and treatment planning. Intraoperative peritoneal lavage collects abdominal fluid to identify not yet visible free cancer cells. The main research gap lies in the poorly defined and potentially insufficient sensitivity of conventional cytology, combined with limited specificity, leading to missed minimal tumor burden and false-negative results. Additionally, peritoneal lavage techniques remain insufficiently standardized, while guideline recommendations vary across tumor entities, with inconsistent indications and tumor-stage definitions. New molecular approaches may improve detection but are not yet widely validated, and standardization may support more individualized treatment strategies.

## 1. Introduction

Peritoneal metastases (PM) are frequently associated with gastrointestinal and gynecological malignancies. The presence of PM typically indicates advanced-stage disease and is associated with poor prognosis. The extent of macroscopic peritoneal tumor spread can be quantified by the Peritoneal Carcinomatosis Index (PCI) described by Sugarbaker and Jacquet [1]. While PCI provides an intraoperative macroscopic assessment of the tumor burden, microscopic dissemination may not be noted visibly. In this context, detection of free malignant cells in peritoneal lavage fluid serves as a diagnostic tool to identify early peritoneal disease before macroscopic carcinomatosis becomes visible. In patients with malignant ascites, analysis of the cellular content can be performed using fluid sampling. However, lack of ascites and/or low cell count can lead to non-detection of tumor cells. Therefore, obtaining adequate material for reliable diagnostic evaluation can be challenging and impair the ability to determine metastatic disease. In gastric adenocarcinoma (GC), detection of malignant cells in peritoneal lavage is classified as stage IV disease according to the Japanese Classification and the Cancer Staging Manual of the American Joint Committee on Cancer [2]. Accurate staging is therefore essential not only for prognostic assessment, but also to avoid unnecessary surgical interventions and offer individual treatment strategies. There are different possibilities of detecting early peritoneal disease. Besides the detection of free cancer cells in peritoneal fluid or peritoneal lavage, quantification of tumor markers, i.e., CEA and CA19-9 can be performed. Furthermore, there is imaging-guided immunofluorescence that can visualize PM. Conventional cytology is the most frequently applied analytical method but demonstrates limited detection rates, especially in patients with minimal tumor burden. Although molecular approaches such as reverse transcription polymerase chain reaction (RT-PCR), one-step nucleic acid amplification (OSNA), and cell-free DNA analysis may improve detection rates, their clinical value is limited by methodological heterogeneity and inconsistent reporting.

Importantly, no international consensus exists regarding key technical aspects of peritoneal lavage, including optimal lavage volume, timing, sampling sites, or analytical methods. This limits comparability between studies and contributes to uncertainty in clinical practice. While previous studies have mainly focused on detection rates and prognostic relevance, a structured evaluation of lavage techniques is lacking.

Therefore, this review provides a comprehensive overview of intraoperative peritoneal lavage techniques, compares detection methods, and evaluates their clinical relevance, with a focus on methodical standardization, rather than solely on detection rates or prognostic implications.

## 2. Materials and Methods

### 2.1. Study Design

This review was conducted as a narrative synthesis of the published literature addressing intraoperative peritoneal lavage for the detection of malignant cells. The primary aim was to provide a comprehensive overview of the current state of research and to integrate relevant findings into a coherent conceptual framework. A formal systematic review methodology based on PRISMA guidelines was not applied, as the focus of this work was on an interpretative and descriptive synthesis rather than on a strictly protocol-driven systematic analysis.

### 2.2. Search Strategy

To identify relevant publications, structured literature research was performed using the PubMed database. The search terms “intraoperative peritoneal lavage” or “peritoneal fluid cytology” were used. Only studies published between 2015 and 2025 were considered in order to ensure the inclusion of recent evidence and contemporary clinical perspectives. This timeframe was selected to capture advances in cytological techniques, evolving diagnostic standards, and current clinical recommendations related to the intraoperative assessment of peritoneal fluid for malignant cells.

### 2.3. Eligibility Criteria

Articles were included if they investigated intraoperative peritoneal lavage for the detection of malignant cells, reported technical details of lavage execution, included patients with gastrointestinal or hepatopancreatobiliary malignancies, and provided data on detection methods, positivity rates, and/or clinical or prognostic relevance. Studies were excluded if they investigated lavage solely as a therapeutic intervention without diagnostic or prognostic intent, lacked a methodological description of lavage technique, or were case reports or conference abstracts without full data. Additional relevant publications cited within eligible studies were screened and included if they fulfilled the predefined criteria.

### 2.4. Selection Process

Two reviewers independently screened titles, abstracts, and subsequently full-text articles to assess their eligibility. Disagreements were solved by discussion and consensus. No automated screening tools were used. Of the 169 publications initially identified, 29 studies were included after the application of the predefined inclusion and exclusion criteria. Extracted data included tumor entity, tumor stage, lavage volume and anatomical site, timing of lavage (pre- or post-resection), sample processing details (e.g., centrifugation), detection method (cytology, immunocytochemistry, molecular assays), reported positivity rates, and clinical and prognostic associations. Studies were grouped according to tumor entity, tumor stage, and analytical methodology. Due to heterogeneity in study design and reporting, we performed a qualitative synthesis rather than a quantitative meta-analysis.

## 3. Results

The majority of the included studies were conducted in Japan [3,4,5,6,7,8,9,10,11,12,13,14,15,16,17,18]. GC was the most frequently investigated tumor entity [10,11,12,13,17,18,19,20,21,22,23,24,25,26,27], followed by PDAC [3,4,5,6,14,15,16,28], CRC [8,9,29,30], and biliary tract cancer (BTC) [7]. Two studies evaluated mixed cohorts comprising various tumor types without providing separate analyses for individual entities [31,32].

### 3.1. Fluid Utilization

All authors performed peritoneal lavage by using differing volumes of physiological saline solution (Table 1). The amount of fluid used ranged from 10 to 1000 mL [3,4,5,6,7,9,10,11,12,13,14,15,16,17,18,19,21,22,23,24,25,26,27,28,29,30,31,32], while two articles did not mention the exact amount used [8,20]. No other solution, i.e., cell culture medium or different crystalloid fluid, was used for free cancer cell detection. There were no specifications regarding the minimum volume of fluid used, nor were there any details provided on the temperature of the fluid prior to performing the lavage.

### 3.2. Timing of Lavage

Regarding the timing of lavage, the majority of the authors sampled peritoneal fluid right after laparotomy or port insertion for laparoscopy [3,4,5,6,7,10,11,12,13,14,15,16,17,18,19,20,21,22,23,24,25,26,27,28,29,30,31] (Table 1). Two publications analyzed the difference in sampling prior to and post tumor resection, seeing no significant difference [8,9]. It was not specified how long the physiological saline solution remained inside the abdominal cavity.

### 3.3. Detection Method

Samples were tested for free cancer cells mostly by conventional staining methods like Hematoxylin-Eosin (HE), Papanicolaou, or Giemsa [3,4,5,7,9,11,12,13,14,15,18,24,30,32] and immunohistochemical staining (IHC) [6]. One article mentioned fluorescence in situ hybridization (FISH) as their detection method [27] (Table 1). No publication used flow cytometry (FACS) for cell detection, despite it being the most commonly used method to analyze immune cells. Besides the cytology testing, there were four publications using reverse transcription polymerase chain reaction (RT-PCR) for RNA detection, especially to detect tumor marker concentration [8,28,31], with one using a double-stranded DNA-KIT (dsDNA-Kit) [15] and another one using a One-Step Nucleic Acid Amplification Assay (OSNA) [23]. The majority of publications reported centrifugation of peritoneal lavage samples prior to cell detection, with centrifugation speeds ranging from 600 [14] to 3000 rpm [17] and with durations of 5 [9,13,20,27,28,29] to 15 min [18]. The most commonly reported setting was 1500 rpm for 5 min [9,13,20,28].

### 3.4. Positive Detection Rate

Comparing the different articles, a wide range of positive detection rates was observed. In all studies, advanced tumor stage was associated with higher detection rates regardless of the lavage technique (Table 2, Table 3 and Table 4). The article by Ji et al. [31] only analyzed patients undergoing HIPEC for various tumor entities (Table 4). All patients presented macroscopic PM, and peritoneal lavage was positive in every case. In addition to assessments of advanced-stage cancer, another publication that included patients undergoing PIPAC showed a detection rate for malignant cells of 57.7% when using cytology [32], while one would usually expect higher rates in malignant ascites. Similar results were seen in different publications, where higher CK19 tumor marker detection rates were recognized in patients with malignant ascites than in patients’ peritoneal lavage fluid [23].

### 3.5. Clinical Relevance of Free Cancer Cell Detection in Peritoneal Fluid

**Figure 1:**
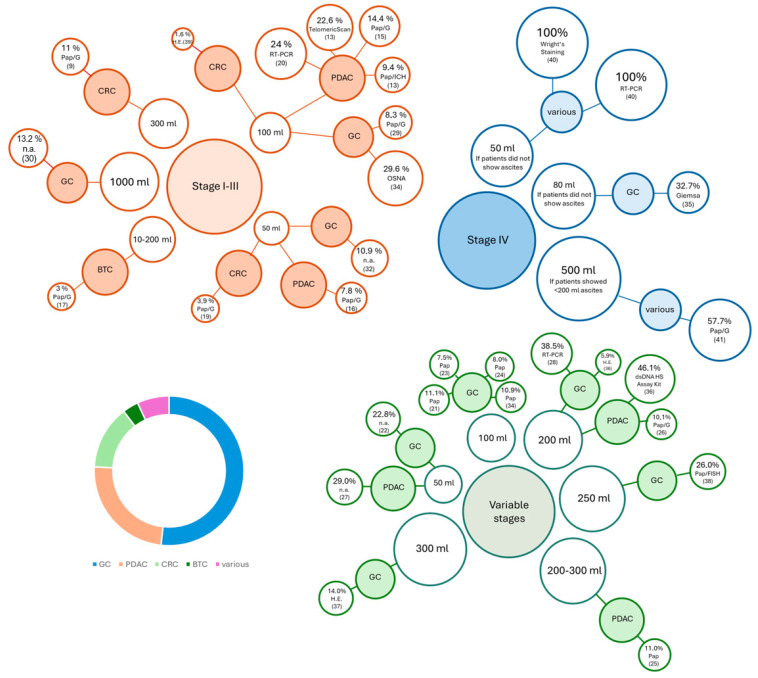
Bubble chart illustrating detection rates of malignant cells in peritoneal lavage (%) across tumor entities stratified by tumor stage (stage I–III, stage IV, variable stages), lavage volume (mL) and tumor entity (gastric adenocarcinoma (GC), pancreatic adenocarcinoma (PDAC), colorectal cancer (CRC), biliary tract cancer (BTC), various). Bubble size corresponds to the detection rate.

In the majority of publications, correlation between positive samples and advanced tumor stage was observed (Figure 1). When samples were taken postoperatively, there was a correlation between higher intraoperative blood loss and increased positive sample rates [33] (Table 2, Table 3 and Table 4). Tumor invasion of the serosa was associated with a higher detection rate of peritoneal cancer cells compared to non-serosal-invading tumors. Tumors with haematogenic metastases also showed higher detection rates than tumors without the same conditions [13].

Among patients with stage I–III disease, most studies identified a significant association between the detection of free malignant cells in peritoneal lavage and poorer overall survival [3,4,5,6,7,9,21,28]. Some studies only showed a small number of patients with positive cytology, which made it challenging to analyze [29]. Sakamoto et al. further demonstrated that positive PCR assays correlated with a higher incidence of local recurrence in rectal cancer [34]. Another study investigating GC revealed that poor histological differentiation or linitis plastica phenotype were more frequently associated with positive cytologic findings in peritoneal lavage [19].

## 4. Discussion

This review highlights the substantial heterogeneity in technical execution, analytical methodology, and reported detection rates of intraoperative peritoneal lavage across tumor entities. Despite its long-standing use in oncological treatment, peritoneal lavage remains insufficiently standardized, which limits comparability between studies and complicates interpretation of clinical relevance.

### 4.1. Technical Variability and Its Impact

One of the most striking findings is the wide variation in lavage protocols. The instilled saline solution volume ranged from 10 to 1000 mL, without consensus regarding optimal quantity. No alternative solutions were used. This variability may significantly influence detection rate, as current evidence from serous fluid cytology suggests that higher volumes may improve detection rates [35]. All studies performed lavage immediately after laparotomy or laparoscopic port insertion to collect representative intraperitoneal material unaffected by iatrogenic tumor manipulation. The two studies comparing pre- and post-resection sampling did not find any significant differences [25], while some patients with initially negative peritoneal lavage cytology showed a positive peritoneal lavage cytology after tumor resection [9]. Based on the above-mentioned findings, manipulation of the tumor and surrounding tissue can affect diagnostic reliability. In particular, iatrogenic dissemination may result in false-positive findings and thereby potentially influence therapeutic decision-making. Consequently, the purpose of peritoneal lavage should determine the timing of its performance. However, critical methodological details, such as dwell time or temperature of the lavage fluid, were not reported. According to experimental evidence that tumor cells rapidly attach to mesothelial surfaces [36], it is possible that even small temporal variations may influence detection rates. The absence of standardized technical parameters represents a major limitation of current practice.

### 4.2. Detection of Malignant Cells: Methods and Limitations

Conventional cytology (HE, Papanicolaou, Giemsa) remains the predominant diagnostic modality [3,4,5,7,9,11,12,13,14,15,18,24,30,32]. Even though these techniques are widely available and cost-effective, their detection rate is limited. IHC and FISH were used only sporadically, while these techniques may provide higher detection rates due to their potential of detecting even small particles. Furthermore, centrifugation protocols showed a wide speed range (600–3000 rpm, 5–15 min), which may affect cell viability. Notably, no study employed flow cytometry (FACS), although this technique offers high-throughput quantitative cellular analysis and could theoretically improve the detection of rare malignant cells.

Molecular approaches, including RT-PCR, OSNA, and dsDNA assays, were used in a minority of studies and generally demonstrated higher detection rates compared to cytology alone [31,32]. PCR-based detection of tumor-markers (e.g., CK19) seemed to identify abdominal tumor burden that was not detectable microscopically. However, increased detection rates have to be evaluated against the uncertain clinical significance of molecular positivity. It remains unclear whether molecular positivity without macroscopic disease uniformly translates into clinically meaningful progression. Prospective validation studies correlating molecular findings with long-term outcomes are therefore essential.

### 4.3. Prognostic Implications

Across all tumor entities, advanced tumor stage correlates with higher positivity rates. Patients with macroscopic peritoneal metastases undergoing HIPEC demonstrated universal cytologic positivity [31], confirming the biological plausibility of lavage-based detection. Interestingly, cytologic detection rates in malignant ascites were higher than in lavage fluid, suggesting that lavage may underestimate tumor burden in the absence of ascites. The relatively moderate positivity rate observed in PIPAC cohorts [32], despite established peritoneal disease, further highlights the limited detection rates of cytology alone. Importantly, most studies reported an association between positive lavage findings and reduced overall survival in stage I–III disease. This suggests that free intraperitoneal tumor cells may represent a marker of biologically aggressive disease rather than merely a staging phenomenon. Nevertheless, the therapeutic consequences of isolated cytologic or molecular positivity remain insufficiently defined.

### 4.4. Clinical Implications and Cost-Effectiveness

The central clinical question is whether peritoneal lavage significantly influences clinical management. In gastric cancer, positive cytology is considered stage IV disease in Japanese guidelines, directly influencing treatment strategy [2]. In contrast, Western guidelines are less explicit and do not show any consensus in terms of performing peritoneal lavage as an implicated diagnostic tool [37].

Model-based economic studies suggest that preoperative or laparoscopic lavage may be cost-effective, preventing non-therapeutic laparotomies [38]. However, such models rely on assumptions regarding test capability and therapeutic benefit that have not been uniformly validated in prospective clinical trials.

### 4.5. Emerging Technologies

Based on the limited detection rates observed with current methods, emerging technologies have been explored to improve detection capability. One promising approach to enhance cell detection is the application of optically induced electrokinetics (OEK) [39], a technique that sorts cells based on their electrical properties, thereby enabling the identification of cancer cells in peritoneal fluid. This method offers several advantages, including high specificity and rapid detection, facilitating timely diagnostic assessment and treatment decisions. Notably, it does not require antibodies, staining, or RNA sequencing and has been reported to be less costly than conventional methods. In the context of testing cell-free DNA analysis in peritoneal lavage, evidence suggests that the quantity of detected DNA correlates with overall survival [40], indicating that this approach may be feasible as a prognostic tool. However, the study is limited by the lack of reported data on sensitivity and specificity. There are limited means to determine whether negative lavage cytology truly excludes the presence of peritoneal tumor cells or merely reflects a false-negative result.

### 4.6. Limitations of This Review

This review has several limitations that should be considered when interpreting the findings. First, it was conducted as a narrative synthesis rather than a fully systematic review following PRISMA guidelines. Although a structured search strategy was applied, the literature search was limited to the PubMed database and predefined search terms, which may have resulted in the omission of relevant studies. In addition, the selection and interpretation of studies may be subject to selection bias and do not provide the same level of reproducibility as a systematic review or meta-analysis.

In addition, substantial heterogeneity was observed across the included studies. Variations in study design, patient populations, and tumor types and stages, as well as differences in the use and timing of neoadjuvant therapy, significantly limit comparability. These factors may influence both the detection rates of malignant cells and their prognostic significance, thereby complicating the interpretation of results across studies. Furthermore, technical aspects of intraoperative peritoneal lavage, such as lavage volume, sampling technique, timing of fluid collection, and cytological processing methods, were frequently reported incompletely or inconsistently. This lack of standardized reporting affects reproducibility and makes it difficult to assess the true impact of methodological differences on diagnostic impact. The overrepresentation of Japanese studies and gastric cancer cohorts may result in a certain degree of selection bias. Nevertheless, this imbalance reflects the current literature as European data remain underrepresented.

### 4.7. Need for Standardization

In summary, these findings indicate that further studies or even an expert-consensus-based recommendation is needed to determine whether peritoneal lavage should be employed as a diagnostic modality and, if so, in which clinical scenarios it should be utilized. It remains unclear whether staging laparoscopy in cT3/T4 gastric cancer should include lavage and whether tumor cell detection should lead to the intensification of systemic therapy, or the consideration of intraperitoneal treatment strategies such as PIPAC. A consensus statement is required to provide a detailed definition of the lavage protocol, including the type of lavage fluid used, the timing of application, the exact site of application, the volume administered, the intra-abdominal dwell time, and the specific analytical techniques employed (e.g., Giemsa or Papanicolaou staining, RNA- or DNA-based assays, flow cytometric analysis, etc.).

## 5. Conclusions

Intraoperative peritoneal lavage represents a potentially valuable tool for detecting occult peritoneal dissemination in gastrointestinal malignancies. Its prognostic relevance has been reported across multiple tumor entities. However, its clinical utility remains limited by heterogeneous techniques, variable timing, and the limited detection rates of conventional cytology. Advanced molecular approaches may improve detection rates and provide additional prognostic information, as suggested by selected studies included in this review. However, their clinical significance and reproducibility require further validation. Given the substantial methodological heterogeneity and limited high-quality evidence, well-designed prospective studies with standardized protocols are essential to define the clinical role of peritoneal lavage. While standardization of lavage parameters may improve comparability, consensus recommendations should be guided by robust clinical evidence. Ultimately, the integration of peritoneal lavage into routine oncologic staging and treatment decision-making will depend on the availability of high-quality data demonstrating its clinical benefit.

## Figures and Tables

**Table 1:** Detection rates of malignant cells in peritoneal lavage fluid, stratified by detection method, tumor entity, anatomical lavage site, and volume of saline solution used for lavage.

**Stage I–III**	**Median (Range) [%]**
Conventional cytology (HE, Papanicolaou, Giemsa)	8.1 (1.6–22.1)
Immunohistochemical staining (IHC)	9.4
RT-PCR (CEA)	16.8 (9.5–24.0)
One-Step NucleicAcid Amplification (OSNA)	29.6
Telomeric Scan	22.6
Tumor entity	
Gastric (GC)	16.0 (8.3–29.6)
Pancreatic (PDAC)	11.9 (7.0–24.0)
Colorectal (CRC)	3.9 (1.6–11.0)
Biliary tract (BTC)	3
Saline solution (mL)	
50	7.8 (3.9–10.9)
100	9.4 (1.6–29.6)
300	11.0
1000	13.2
Location of fluid application	
Tumor area	16.6 (11.0–22.1)
Subhepatic space	13.5 (3.0–24.0)
Ubiquitous	8.3 (7.0–18.8)
Pelvic cavity	7.8 (3.0–14.4)
**Stage IV**	**median (range of detected tumor cells) [%]**
Conventional cytology (H.E., Papanicolaou, Wright’s staining, Giemsa)	57.7 (32.7–100)
RT-PCR	100
Tumor entity	
GC	32.7
Various	100 (57.7–100)
Saline solution(mL)	
50	100
80	32.7
500	57.7
Location of fluid application	
Abdominal cavity	78.9 (57.7–100)
Epigastrium	32.7
**Tumor stage not specified**	**median (range) [%]**
Conventional cytology (H.E., Papanicolaou, Giemsa)	11.0 (10.1–26.0)
FISH	26.0
RT-PCR	38.5
dsDNA HS Assay	46.1
Tumor entity	
GC	11.1 (7.5–38.5)
PDAC	29.0 (11.0–46.1)
Saline solution (mL)	
50	25.9 (22.8–29.0)
200	38.5 (5.9–46.1)
300	14
Location of fluid application	
Pelvic cavity	11.0 (7.5–38.5)
Ubiquitous	25.0 (5.9–26)
Abdominal cavity, collected from Douglas	46.1

**Table 2:** Selected publications, listed by authors and country, assessed patients that received peritoneal lavage (*n*), patients with cell detection in peritoneal lavage (*n*, %), time of peritoneal lavage (p-lap—post laparotomy; p-lasc—post laparoscopic port insertion; prtr—prior tumor resection; ptr—post tumor resection; n.a.—not available), fluid used, timing of intra-abdominal fluid application, location of applied fluid, variable stages (Stage I–III), and no clinical distant metastases.

Article	Primary Tumor	*N*	*n* Positive (%)	Time of Lavage	Volume of Wash Fluid (Saline Solution)	Site of Lavage	Cell Detection Method
**Cytology**							
Koganti et al. 2016, India [18]	GC	60	5 (8.3%)	p-lap	100 mL	subphrenic spaces, pelvic cavity	Papanicolaou and Giemsa
Ikoma et al. 2016, USA [19]	GC	514	68 (13.2%)	n.a.	1000 mL	n.a.	n.a.
Zhang et al. 2017, China [20]	GC	136	30 (22.1%)	p-lap	n.a.	tumor and surrounding tissue	HE
Young et al. 2022, USA [21]	GC	55	6 (10.9%)	n.a.	50 mL	three abdominal quadrants	n.a.
Yin et al. 2024, China [22]	GC	85	16 (18.8%)	p-lap	500 mL	around transverse colon and stomach, collected around stomach, spleen and liver	microscope
Sakoda et al. 2021, Japan [3]	PDAC	440	30 (7.0%)	p-lap	100 mL	pelvic cavity	Papanicolaou and Giemsa
Mashiko et al. 2023, Japan [4]	PDAC	97	14 (14.4%)	p-lap	100 mL	abdominal cavity	Papanicolaou and Giemsa
Ishida et al. 2023, Japan [5]	PDAC	231	18 (7.8%)	p-lap	50 mL	pelvic cavity	Papanicolaou and Giemsa
Tanemura et al. 2024, Japan [6]	PDAC	53	5 (9.4%) vs. 12 (22.6%)	p-lap	100 mL	pelvic cavity	Papanicolaou/IHC vs. TelomericScan
Matsukuma et al. 2021, Japan [7]	BTC	169	5 (3%)	p-lap	10–200 mL	pelvic and/or subhepatic cavity	Papanicolaou and Giemsa
Salamanca et al. 2020, Spain [29]	CRC	188	2 (1.6%)	p-lap/ p-lasc	100 mL	n.a.	HE
Shalaby et al. 2021, Egypt [30]	CRC	90	10 (11%)	p-lap	300 mL	around the tumor, paracolic gutter	Papanicolaou and Giemsa
Ouchi et al. 2022, Japan [8]	CRC	95	2 (2.1%)	prtr and ptr	n.a.	n.a.	n.a.
Kobayashi et al. 2024, Japan [9]	CRC	1378	54 (3.9%)	prtr and ptr	50 mL	pelvic cavity	Papanicolaou and Giemsa
**mRNA**							
Takahashi et al. 2018, Japan (CEA) [28]	PDAC	237	57 (24%)	p-lap	100 mL	subhepatic space	RT-PCR
Gęca et al. 2021, Poland (CK19) [23]	GC	27	7 (29.6%)	p-lap	100 mL	n.a.	One-Step Nucleic Acid Amplification (OSNA) Assay
Ouchi et al. 2022, Japan (CEA) [8]	CRC	95	9.50%	prtr and ptr	n.a.	n.a.	RT-PCR

**Table 3:** Selected publications, listed by authors and country, assessed patients that received peritoneal lavage (*n*), patients with cell detection in peritoneal lavage (*n*, %), time of peritoneal lavage (p-lap—post laparotomy; prtr—prior tumor resection), fluid used, timing of intra-abdominal fluid application, location of applied fluid, and variable stages (Stage IV).

Article	Primary Tumor	*N*	*n* Positive (%)	Time of Lavage	Volume of Wash Fluid (Saline Solution)	Site of Lavage	Cell Detection Method
**Cytology**							
Hoskovec et al. 2017, Czech Republic [24]	GC	49	16 (32.7%)	p-lap	80 mL (if no ascites present)	epigastric area	Giemsa
Ji et al. 2016, China [31]	various	50	50 (100%)	p-lap	50 mL (if no ascites present)	abdominal cavity	Wright’s staining
Graversen el at. 2019, Denmark [32]	various	26	15 (57.7%)	prtr	500 mL (if patient had less than 200 mL of ascites)	abdominal cavity	Papanicolaou, Giemsa, HE
**mRNA**							
Ji et al. 2016, China [31]	various	50	50 (100%)	p-lap	50 mL (if no ascites present)	abdominal cavity	RT-PCR

**Table 4:** Selected publications, listed by authors and country, assessed patients that received peritoneal lavage (*n*), patients with cell detection in peritoneal lavage (*n*, %), time of peritoneal lavage (p-lap—post laparotomy; p-lasc—post laparoscopic port insertion), fluid used, timing of intra-abdominal fluid application, location of applied fluid, and variable stages.

Article	Primary Tumor	*N*	*n* Positive (%)	Time of Lavage	Volume of Wash Fluid (Saline Solution)	Site of Lavage	Cell Detection Method
**Cytology**							
Kano et al. 2015, Japan [11]	GC	1039	116 (11.1%)	p-lap/ p-lasc	100 mL	abdominal and pelvic cavity	Papanicolaou
Okabe et al. 2015, Japan [10]	GC	123	28 (22.8%)	p-lasc	50 mL	pelvic cavity and left subphrenic space	n.a.
Higaki et al. 2017, Japan [12]	GC	911	68 (7.5%)	p-lap	100 mL	pelvic cavity	Papanicolaou
Sakata et al. 2019, Japan [13]	GC	973	78 (8.0%)	p-lap	100 mL	right subdiaphragmal space, Douglas	Papanicolaou
Hasbahceci et al. 2018, Turkey [25]	GC	34	2 (5.9%)	p-lap	200 mL	ubiquitous	HE
Gęca et al. 2021, Poland [23]	GC	55	6 (10.9%)	1. p-lasc 2. p-lap 3. surgery	100 mL	abdominal cavity	n.a.
Cieśla et al. 2021, Poland [26]	GC	100	14 (14%)	p-lap/ p-lasc	300 mL	cancer area	HE
Xuan et al. 2024, China [27]	GC	50	13 (26.0%)	p-lasc	250 mL	ubiquitous	Papanicolaou FISH
Hirabayashi et al. 2015 Japan [14]	PDAC	162	18 (11%)	p-lap	200–300 mL	pelvic cavity	Papanicolaou
Suenaga et al. 2021, Japan [15]	PDAC	89	9 (10.1%)	p-lap	200 mL	abdominal cavity, collected from Douglas pouch	Papanicolaou, Giemsa
Takadate et al. 2021, Japan [16]	PDAC	146	42 (29.0%)	p-lasc	50 mL	n.a.	n.a.
**RT-PCR**							
Nakanishi et al. 2019, Japan [17]	GC	187	72 (38.5%)	p-lap	200 mL	pelvic cavity	RT-PCR
**dsDNA HS Assay Kit**							
Suenaga et al. 2021, Japan [15]	PDAC	89	41 (46.1%)	p-lap	200 mL	abdominal cavity, collected from Douglas pouch	dsDNA HS Assay Kit

## Data Availability

No new data were created in this review. Publicly available data was analyzed.

## Abbreviations

The following abbreviations are used in this manuscript:

BTC	biliary tract cancer
CRC	colorectal cancer
CRS	cytoreductive surgery
FISH	fluorescence in situ hybridization
GC	gastric adenocarcinoma
HE	Hematoxylin-Eosin
HIPEC	hyperthermic intraperitoneal chemotherapy
IHC	immunohistochemical staining
OEK	optically induced electrokinetics
PCI	Peritoneal Carcinomatosis Index
PDAC	pancreatic adenocarcinoma
PIPAC	pressurized intraperitoneal aerosol chemotherapy
p-lap	post laparotomy
p-lasc	post laparoscopic port insertion
PM	Peritoneal metastases
PRISMA	Preferred Reporting Items for Systematic Reviews and Meta-Analyses
prtr	prior tumor resection
ptr	post tumor resection
RT-PCR	reverse transcription polymerase chain reaction
